# Identification and characterization of distinct IL-17F expression patterns and signaling pathways in chronic lymphocytic leukemia and normal B lymphocytes

**DOI:** 10.1007/s12026-015-8722-5

**Published:** 2015-10-19

**Authors:** Barbara Sherry, Preetesh Jain, Pui Yan Chiu, Ling Leung, Steven L. Allen, Jonathan E. Kolitz, Kanti R. Rai, Jacquie Barrientos, Spencer Liang, Rachael Hawtin, Nicholas Chiorazzi

**Affiliations:** Center for Immunology and Inflammation, The Feinstein Institute for Medical Research, 350 Community Drive, Manhasset, NY 11030 USA; Karches Center for Chronic Lymphocytic Leukemia Research, The Feinstein Institute for Medical Research, Manhasset, NY USA; Department of Medicine, Hofstra North Shore-LIJ School of Medicine, Hofstra University, Hempstead, NY USA; Department of Molecular Medicine, Hofstra North Shore-LIJ School of Medicine, Hofstra University, Hempstead, NY USA; Department of Leukemia, The University of Texas MD Anderson Cancer Center, Houston, TX USA; Department of Internal Medicine, University of Texas Medical School at Houston, Houston, TX USA; Nodality, Inc., South San Francisco, CA USA; Department of Medicine, North Shore University Hospital and Long Island Jewish Medical Center, North Shore-LIJ Health System, Manhasset, NY USA

**Keywords:** Chronic Lymphocytic Leukemia (CLL), Interleukin-17 (IL-17), Th17 cells

## Abstract

Chronic lymphocytic leukemia (CLL) is characterized by a progressive accumulation of B lymphocytes. T cell abnormalities are a common feature of CLL and contribute to impaired immune function in these patients. T cells are ineffective in eliminating the leukemic clone and may actually promote tumor growth and survival. Previous work from our laboratory documented elevated circulating levels of IL-17A-producing Th17 cells in CLL patients as compared to healthy age-matched control subjects. These high circulating Th17 levels associated with better prognostic markers and significantly longer overall survival, even among patients whose clones used unmutated *IGHV*s (U-CLL). Recent studies suggest that Th17 cells are heterogeneous, expressing different profiles of cytokines, and that different subsets of Th17s mediate different biological functions. In the present study, we found significantly higher levels of IL-17F-expressing CD4^+^ T cells in CLL versus healthy peripheral blood mononuclear cells following in vitro stimulation in the presence of Th17-promoting cytokines. Furthermore, the differentiation of IL-17F-expressing Th17 cells was significantly enhanced when purified CD4^+^ T cells from CLL patients were cultured in the presence of autologous CLL B cells. Lastly, single-cell network profiling revealed that IL-17F triggers NFκB phosphorylation in T and B cells from patients with CLL, but not age-matched healthy controls. Taken together, our data suggest that the phenotype of Th17 cells in CLL patients is distinct from healthy individuals, expressing higher levels of IL-17F, and that B and T cells from CLL patients are particularly responsive to IL-17F, as compared to healthy age-matched control individuals.

**Electronic supplementary material:**

The online version of this article (doi:10.1007/s12026-015-8722-5) contains supplementary material, which is available to authorized users.

## Introduction

Chronic lymphocytic leukemia (CLL), the most common adult leukemia in the USA, is characterized by the progressive accumulation of CD5^+^ B lymphocytes [1, 2]. Growth and survival of the leukemic B cell clone are regulated by microenvironmental elements present within the bone marrow and secondary lymphoid tissues [3]. CLL cells receive critical antigen-triggered signals through the B cell receptor (BCR) as well as regulatory input from a range of accessory cells. These include T cells, stromal cells, endothelial cells, follicular dendritic cells, and macrophages that interact directly with leukemic B cells via a complex set of adhesion molecules and cytokines [3]. Within lymphoid tissues, T cells, mainly activated CD4^+^ cells, reside in proliferation centers and influence CLL growth and survival, presumably via CD40L–CD40 interactions [4]. In addition to surface interactions, T cells secrete an array of soluble factors that contribute to CLL cell survival and proliferation, including IL-4, IL-2, TNFα, and IFN. The requirement for T cells in CLL growth and survival is supported by data from a xenograft model of CLL where engraftment and proliferation of primary leukemic B cells are dependent upon the presence of activated T cells [5].

T cell dysregulation is a common feature of CLL and is thought to contribute to increased susceptibility to bacterial and opportunistic infections in this population [6, 7]. In CLL, leukemic B cells and/or the leukemic microenvironment have been reported to trigger multiple T cell defects including impaired formation of an immunologic synapse, altered expression of co-stimulatory molecules, and impaired cytotoxic killing of autologous tumors [8–10]. The dysregulating influence of CLL cells themselves and the CLL microenvironment on the T cell compartment is supported by studies showing a genetic signature for in vitro-stimulated CD4^+^ cells from patients with CLL consistent with downregulation of T cell receptor signaling cascades and reduced cytokine secretion [11]. The balance of T cell subsets is also skewed in the context of CLL [7, 12–14]. Levels of regulatory T cells (Tregs), which are known to suppress host antitumor immune responses, have been reported to correlate with aggressive disease and time-to-first treatment (TTFT) in patients with CLL [15–17]. In addition, we and others have demonstrated that Th17 cells, a subset of T cells with inflammatory properties, are elevated in CLL and correlate with better outcome [16, 18].

Th17 cells are generated from naïve T cells when they are activated in the presence of a select set of cytokine signals that include IL-1β, IL-6, IL-21, IL-23, and TGFβ [38]. The early observation of genetic mutations in STAT3 in patients with hyperimmunoglobulinemia E (Job’s) syndrome [19] suggested a role for this transcription factor in the differentiation of Th17 cells. A large body of work now supports STAT3 as a driving force in T cell differentiation into Th17, inducing the transcription of IL-17A and the Th17-specific transcription factor, retinoic acid receptor-related orphan nuclear receptor (RORγt). IL-6 and IL-21, two cytokines known to induce the differentiation of naïve CD4^+^ T cells into Th17 cells, activate the JAK/STAT pathway, suggesting that STAT3 activation may be a common mechanism through which these cytokines regulate Th17 differentiation [20]. Further supporting a central role for STAT3 in Th17 differentiation is the observation that Th17 differentiation is prevented in CD4^+^ T cells transfected with STAT3 siRNA [20].

Th17 cells have been documented in several human cancers including CLL and found to play pro- and antitumor roles depending on the tumor [21–25]. While the specific elements within the CLL microenvironment that promote Th17 generation are currently unknown, we have shown that two cytokines critical for Th17 differentiation in healthy individuals, IL-1β and IL-6, are elevated in a subset of CLL patients and belong to a cluster of cytokines whose presence correlates with longer TTFT and longer overall survival in this disease [26]. This finding, taken together with our observation that Th17 levels are higher in CLL patients with better prognosis, suggests that Th17 cells and cytokines that promote the development of Th17s favor superior outcome in CLL.

However, the extent that IL-17A itself, or another one or more of the cytokines released by Th17 cells (e.g., IL-17F), exerts antitumor activity in this disease is not known. IL-17A and IL-17F have many overlapping biological activities [27], but recent studies indicate that the two can mediate discrete functions as well [28–30]. Both IL-17A and IL-17F bind to the same heterodimeric receptors, IL-17RA and IL-17RC, but with different affinities, which may explain in part the overlapping and divergent activities of these structurally very similar proteins [31]. In addition to differences in their bioactivity profiles, IL-17A and IL-17F can be differentially regulated at the cellular and tissue level, creating another layer of complexity. To date, Th17 cells in CLL patients have only been characterized with respect to their expression of IL-17A; whether they express and/or are responsive to IL-17F is not known.

Here, we report that levels of IL-17F produced by cytokine activated Th17 cells derived from CLL patients are significantly higher compared to healthy controls. Furthermore, both leukemic B cells and CLL T cells are responsive to IL-17F-mediated signaling, specifically activating NFkB, whereas B and T cells from age-matched healthy controls are not. Taken together, our data suggest that in tissue microenvironments sustaining the activation and division of leukemic B cells, the functional program of Th17s in CLL might be distinct from that seen in healthy subjects, favoring IL-17F production.

## Materials and methods

### Study population

Seventy-four CLL patients identified according to the International Workshop on CLL diagnostic criteria [32] and 28 age-matched healthy volunteers participated in these studies. Supplementary Table 1 reports clinical and laboratory characteristics of patients and healthy age-matched control subjects and indicates which studies were performed with cells or serum from each CLL patient and healthy subject. All CLL patients were untreated. All procedures in this study (IRB# 08-202A) were in accordance with the ethical standards of The North Shore—Long Island Jewish Health System Office of the Institutional Review Board, and informed consent was obtained from all study subjects.

### Blood collection and processing

Peripheral blood mononuclear cells (PBMCs) from CLL patients and healthy subjects were isolated from whole blood by density gradient centrifugation using Ficoll-Paque (GE Healthcare, Piscataway, NJ) and were frozen in fetal calf serum plus 10 % dimethylsulfoxide (DMSO) in liquid N_2_ (Sigma-Aldrich, St Louis, MO) for analysis at a later point. For ex vivo (baseline/pre-stimulation) flow cytometric analyses of Il-17F and IL-17A, frozen cells were thawed and stained for surface phenotyping markers and intracellular IL-17F and IL-17A as described.

### Long-term cell culture

Frozen PBMCs from CLL and healthy subjects were thawed and cultured for 7 days in 24-well plates pre-coated for 2 h with 1 µg/ml anti-CD3 mAb (eBioscience Inc., San Diego, CA) at a cell density of 1 × 10^6^ cells per ml in RPMI1640 medium supplemented with 10 % NHS (normal human serum), 1 μg/ml anti-CD28 mAb (Beckman Coulter, Indianapolis, IN), and a Th17-promoting cytokine cocktail containing recombinant human IL-1β, IL-6, and IL-23 (R&D Systems, Minneapolis, MN; 10 ng/ml final concentration for all). At day 7, non-adherent cells were collected, washed, and stained for surface markers and intracellular IL-17F and IL-17A. For T ± CLL B cell co-culture studies, frozen PBMCs from CLL patients (*n* = 3) were thawed, and a portion of cells from each patient was used for the isolation of CLL B cells using a B-CLL Cell Negative Isolation Kit (Miltenyi Biotec Inc., San Diego, CA). The remaining cells from each patient were used for the isolation of CD4^+^ T cells using a CD4^+^ Cell Positive Isolation Kit (Miltenyi Biotec Inc.). Cell purifications were performed according to manufacturer’s protocols. Purified CD4^+^ T cells (1 × 10^5^ cells/ml) were cultured for 7 days on anti-CD3/CD28-coated 96-well microplates alone or together with purified autologous CLL B cells at a B:T ratio of 10:1. Various combinations of Th17-promoting cytokines (10 ng/ml) were added to wells as indicated. Each condition was set up in triplicate. At day 7, supernatants were collected and analyzed for IL-17F and IL-17A by ELISA.

### Flow cytometric analysis of IL-17A and IL-17F in PBMCs ex vivo and after 7-day stimulation with anti-CD3/C28 mAbs

For detection of intracellular IL-17A and IL-17F, freshly thawed PBMC suspensions from CLL patients and healthy subjects were stimulated for 5 h with 10 ng/ml phorbol 12-myristate 13-acetate (PMA) and 250 ng/ml ionomycin in the presence of monensin (BD Biosciences, San Jose, CA). Stimulated cells were centrifuged at 1200 g for 7 min at 10 °C, washed, and then surface stained by incubating cells (1–2 × 10^6^/ml) for 30 min at room temperature in the dark with antihuman mAbs: anti-CD3-APC-H7, anti-CD4-APC, anti-CD8-PerCP (all BD Biosciences), and anti-CD161-PerCPCy 5.5 (Biolegend, San Diego, CA). Cells were subsequently fixed and permeabilized using Cell Fixation/Permeabilization Kit (BD Biosciences) and stained intracellularly with antihuman IL-17F PE (eBioscience Inc.) or IL-17A AlexaFluor 488 (eBioscience Inc.) according to manufacturer’s instructions. Samples were acquired on an LSR II (BD Biosciences) and analyzed using FlowJo software (version 8.8.6). IL-17F- and IL-17A-expressing CD4^+^ T cells in CLL and healthy PBMC cultures activated for 7 days in vitro under Th17 polarizing conditions were similarly analyzed.

### ELISA analyses

IL-17F and IL-17A levels were determined in supernatant fluids using commercially available DuoSet ELISA kits (R&D Systems) according to manufacturer’s directions. IL-23 levels in stored, frozen serum samples from CLL patients (*N* = 57) and age-matched normal healthy (*N* = 15) control subjects were determined using a commercially available ELISA kit (R&D Systems) according to manufacturer’s directions. All analyses were carried out in duplicate. Statistical significance was examined by the Mann–Whitney test (comparison of ex vivo and in vitro-stimulated IL-17F levels in CLL and healthy PBMCs) or two-way ANOVA (T ± CLL B cell co-culture studies).

### Single-cell network profiling (SCNP) assay

SCNP, a multiparametric, single-cell flow cytometry analysis approach [33–35], was used to analyze the responsiveness of cryopreserved PBMCs from 13 CLL patients and four healthy donors. Responsiveness of CLL and healthy B and T cells to modulators including IL-17F, IL-17A, IL-6, and IL-1β (R&D Systems) was quantified simultaneously across multiple nodes (a node being an extracellular modulator combined with a specific intracellular readout, e.g., IL-17 → pNFkBp105) in multiple immune cell subsets including CD4^+^CD3^+^ and CD4^−^CD3^+^ (CD8) T cells as well as CD19^+^ B cells in healthy donors and CD19^+^CD5^+^ cells in CLL patient samples. SCNP is performed without cell subset isolation, thereby allowing the examination of functional cell signaling in individual cells and cross-talk among different cells [33]. Cells were incubated in 96-well plates with 100,000 cells per well and either unmodulated (capturing basal signaling) or modulated with the abovementioned cytokines. Cells were fixed, permeabilized and incubated with a cocktail of fluorochrome-conjugated antibodies that recognize extracellular lineage markers and intracellular epitopes. Evoked intracellular signaling in the NFkB (p-NFkBp105), PI3K (p-AKT), MAPK (p-ERK), and STAT (p-STAT1, p-STAT3, pSTAT5) pathways was quantified at 5 min post-stimulation.

### Statistical analyses

Group comparisons were carried out using the Mann–Whitney test, Wilcoxon matched-pairs signed rank test, unpaired two-sided Student’s *t* test, or two-way analysis of variance (ANOVA), as appropriate. Pairwise comparisons were carried out using a Bonferroni-type adjustment (*p* < 0.01). All statistical analyses were performed by GraphPad Prism version 6.0 for Windows (GraphPad Software, San Diego, CA). In all cases, *p* < 0.05 was accepted as significant.

## Results

### Ex vivo levels of Th17F cells are higher in CLL PBMC as compared to healthy controls

**Fig. 1:**
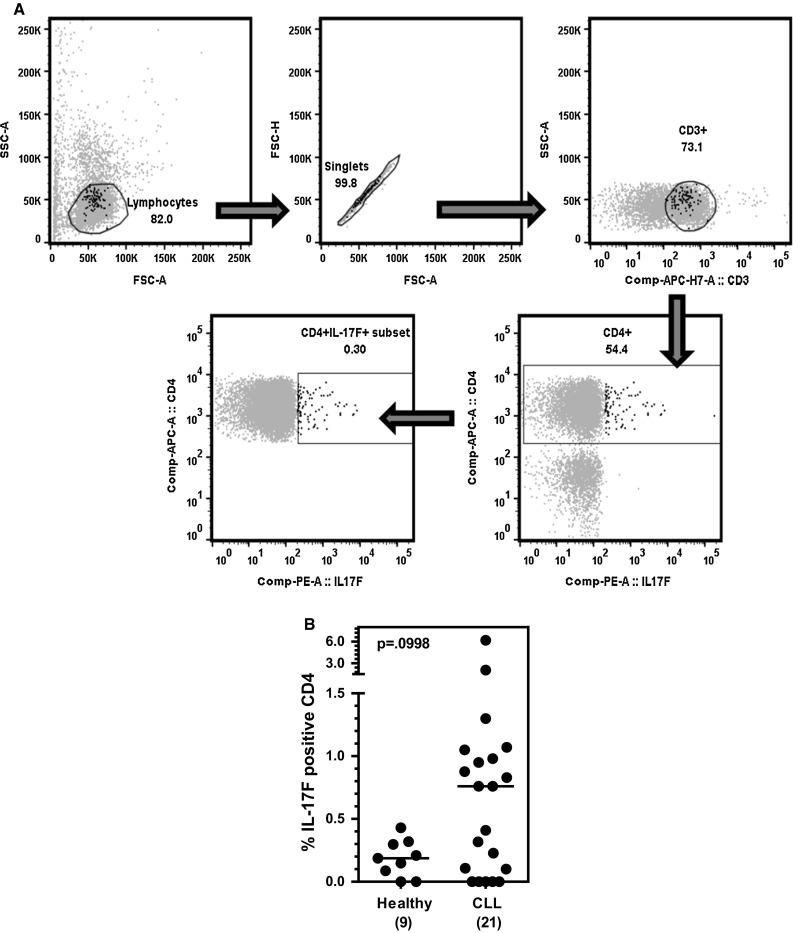
Percentage of IL-17F-expressing Th17 cells in the blood of CLL patients versus healthy controls. **a** *Flow cytometry strategy to quantify IL*-*17F*-*expressing CD4* ^+^ *T cells* (“*Th17F*”). PBMCs from a single age-matched healthy control subject were stained for surface markers and intracellular IL-17F and analyzed by flow cytometry. Plots illustrate gating of the terminally selected “Th17F” (CD3^+^CD4^+^IL-17F^+^) population (*lower left plot*). Back-gating shows that the “Th17F” population (*black highlighted cells in all plots*) selected for analysis is comprised of singlet lymphocytes (*upper left and middle plots*) that are CD3^+^ (*upper right plot*) and CD4^+^ (*lower right plot*). **b** Frozen aliquots of PBMCs from CLL patients (*n* = 21) and healthy age-matched individuals (*n* = 9) were thawed and stained for surface markers and intracellular IL-17F (or IL-17A) and analyzed by flow cytometry. Statistical significance was examined by the Mann–Whitney test

Th17 cells are classically defined by expression of IL-17A, but they can produce IL-17F as well. While IL-17A and IL-17F are highly homologous and exert some overlapping biological activities, they are distinct molecules with a number of discrete functions. We compared the expression of IL-17F in circulating CD4^+^ T cells from CLL patients (*n* = 21) and age-matched healthy controls (*n* = 9) at baseline by intracellular flow cytometry using the strategy outlined in Fig. 1a. The percentage of IL-17F-expressing CD4^+^ T cells trended higher in CLL patients as compared to healthy age-matched individuals, but the difference did not reach statistical significance (*p* = 0.099) (Fig. 1b).

### IL-23 levels are significantly higher in CLL than control sera from normal donors

**Fig. 2:**
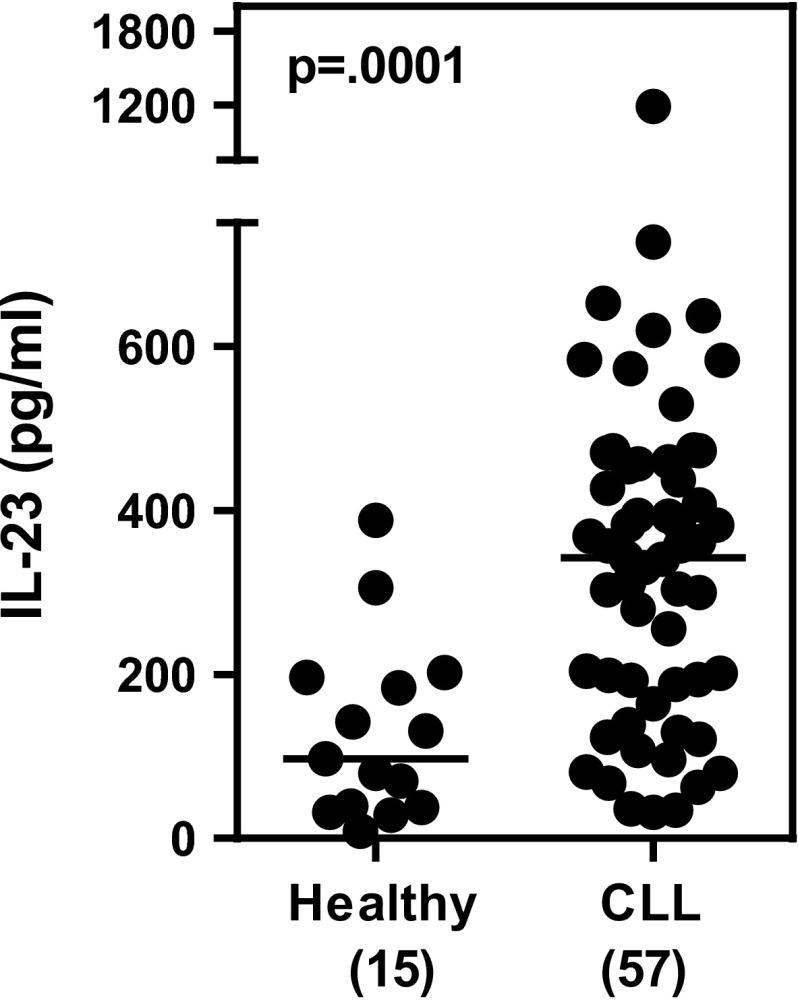
IL-23 levels are significantly higher in CLL versus control sera. Serum samples from CLL patients (*N* = 57) and age-matched normal healthy control subjects (*N* = 15) were analyzed for IL-23 by sandwich ELISA. Serum levels of IL-23 were significantly higher in CLL patients (*p* = 0.0001) compared with age-matched control subjects. Statistical significance was examined by the Mann–Whitney test

Our finding that ex vivo levels of Th17F cells trended higher in CLL PBMCs as compared to healthy controls suggests the presence within the CLL microenvironment of elements (e.g., cytokines) that favor Th17 cell differentiation. Cytokines known to induce the differentiation and/or expansion of Th17s include IL-1β, IL-6, and IL-23, among others [36–38]. Our previous work revealed that circulating levels of IL-1β and IL-6 are elevated in a subset of CLL patients and that these two cytokines belong to a cluster of cytokines whose presence correlates with longer TTFT and overall survival in the CLL patient population [26]. To determine whether levels of IL-23 were also elevated in the context of CLL, we measured the levels of this cytokine in serum samples collected from CLL patients (*N* = 57) and age-matched healthy donors (*N* = 15) by sandwich ELISA. Serum levels of IL-23 were significantly elevated in CLL patients as compared with healthy controls (*p* = 0.0001) (Fig. 2). Considerable heterogeneity was observed in the serum IL-23 levels in different CLL patients. Whether serum IL-23 levels correlate with specific prognostic markers (e.g., mutational status, CD38 expression) remains to be determined.

### CLL PBMCs exhibit higher IL-17F-expressing Th17 cells after 7 days in vitro under activating conditions than PBMCs from healthy donors

**Fig. 3:**
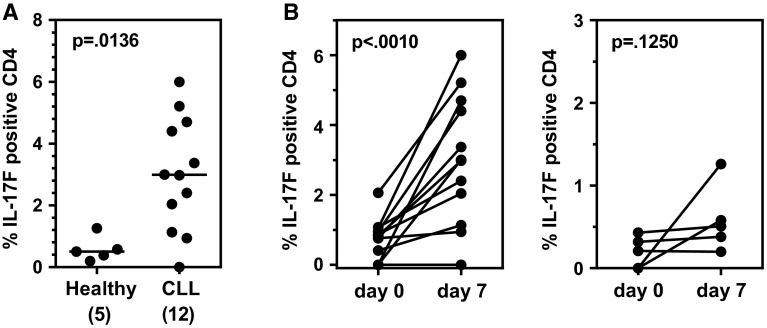
CLL PBMCs exhibit higher IL-17F-expressing Th17 cells after 7 days in vitro activation under Th17-polarizing conditions than PBMCs from healthy donors. **a** PBMCs from CLL patients (*n* = 12) and healthy age-matched control subjects (*n* = 5) were stimulated in vitro with anti-CD3/CD28 mAbs for 7 days in the presence of a Th17-promoting cocktail (IL1 + IL23). At day 7, cells were collected, stained for surface markers and intracellular IL-17F (or IL-17A) and analyzed by flow cytometry. Statistical significance was examined by the Mann–Whitney test. **b** Comparison of IL-17F-expressing CD4^+^ T cell percentages for individual CLL patients (*n* = 12;* left panel*) and age-matched healthy subjects (*n* = 5;* right panel*) pre- and post-stimulation by TCR cross-linking for 7 days in the presence of a Th17-promoting cocktail (IL1 + IL23). Statistical significance was examined by the Wilcoxon matched-pairs signed rank test

We next analyzed the percentages of CD4^+^ IL-17F-expressing cells in CLL (*n* = 12) and healthy (*n* = 5) PBMCs activated in vitro by TCR cross-linking. When PBMCs were activated with anti-CD3/CD28 for 7 days in culture in the presence of a cocktail of Th17-promoting cytokines (Fig. 3a), the percentages of CD4^+^ T cells expressing IL-17F were significantly higher for CLL as compared to control PBMCs (*p* = 0.0136). When PBMCs were activated for 7 days in the absence of exogenously added Th17-promoting cytokines, no significant difference between baseline and day 7 percentages was observed for either CLL (*p* = 0.5438) or healthy (*p* = 0.4258) (data not shown). In a subsequent analysis, we compared the percentage of IL-17F-expressing CD4^+^ T cells at baseline and after activation in the presence of Th17-promoting cytokines for each patient (Fig. 3b, left panel) and healthy donor (Fig. 3b, right panel). In all CLL patients with the exception of one, the percentage of IL-17F-expressing CD4^+^ T cells was higher after in vitro activation in the presence of the Th17-promoting cytokine cocktail, with the positive change between baseline and day 7 being statistically different (*p* = 0.0010). For age-matched healthy subjects, the percentage of CD4^+^ T cells expressing IL-17F was not statistically different between baseline and day 7 (*p* = 0.1250). A similar comparison was performed for IL-17A, and the percentage of IL-17A-expressing CD4^+^ T cells in CLL PBMCs (*n* = 12) at baseline was not statistically different from the percentage observed after cells were activated with anti-CD3/CD28 for 7 days in either the presence or absence of Th17-promoting cytokines (*p* = 0.8125 and 0.9548, respectively; data not shown).

### CLL B cells enhance the generation of IL-17F-producing Th17s in vitro

**Fig. 4:**
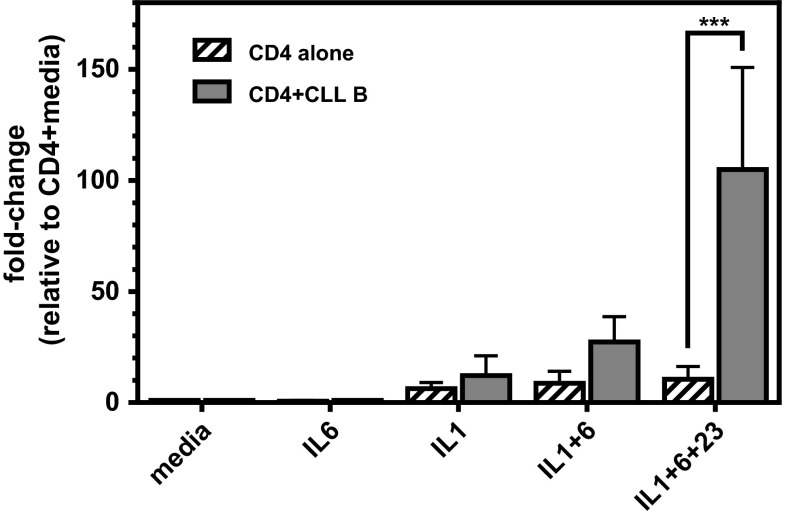
Addition of autologous CLL B cells to purified CD4^+^ T cells enhances production of IL-17F. Purified CD4^+^ T cells from a subset of CLL patients (*n* = 3) were cultured for 7 days on anti-CD3/CD28-coated plates alone or together with purified autologous CLL B cells at a B:T ratio of 10:1 and various Th17-promoting cytokine combinations (IL-6, IL-1β, IL-1β + IL-6, IL-6 + IL-1β + IL-23; all cytokines at 10 ng/ml). At day 7, supernatants were collected and analyzed for IL-17F by ELISA. Data are presented as fold change relative to CD4^+^ T cell only cultures (no cytokine additions). Comparisons were made using two-way ANOVA followed by Bonferroni post hoc tests to compare effects of addition of CLL B cells and various cytokine treatments. (***p = 0.001)

Since the previous set of experiments was performed with whole PBMC cultures, the higher percentages of IL-17F-expressing CD4^+^ T cells observed in CLL PBMCs could reflect an inherent difference in the CLL T cells themselves or a modulatory effect mediated by autologous CLL B cells or another immune cell present in the CLL PBMC population. To begin to directly assess whether CLL B cells promote T cell differentiation to IL-17F-producing Th17s, purified CD4^+^ T cells from three independent CLL patients were cultured alone or together with autologous CLL B cells for 7 days in the presence (or absence) of various combinations of Th17-polarizing cytokines (IL-6, IL-1β, IL-1β + IL-6, IL-6 + IL-1β + IL-23). IL-17F levels in the supernatant fluid at day 7 were higher when CD4^+^ T cells were co-cultured with CLL B cells as compared to when CD4^+^ T cells were cultured alone (Fig. 4). A two-way ANOVA indicated that the enhancement observed upon addition of CLL B cells was significant (*p* = 0.0242). Post hoc analyses employing Bonferroni correction revealed that the enhancing effect of CLL B cells on CD4^+^ T cell IL-17F production was significant only in cultures differentiated in the presence of IL-6 + IL-1 + IL-23 (*p* = 0.0017). A significant difference in IL-17F levels was also observed in cultures treated with different combinations of Th17-inducing cytokines (*p* = 0.0079). Lastly, a significant interaction effect (*p* = 0.0287) was observed between the addition of CLL B cells and the particular Th17-promoting cytokine(s) added indicating that the stimulatory effect of CLL B cells on IL-17F production by CD4^+^ T cells is dependent upon the specific cytokine cocktail used to trigger Th17 differentiation.

### IL-17F triggers increased NFkB signaling in CLL B cells

**Fig. 5:**
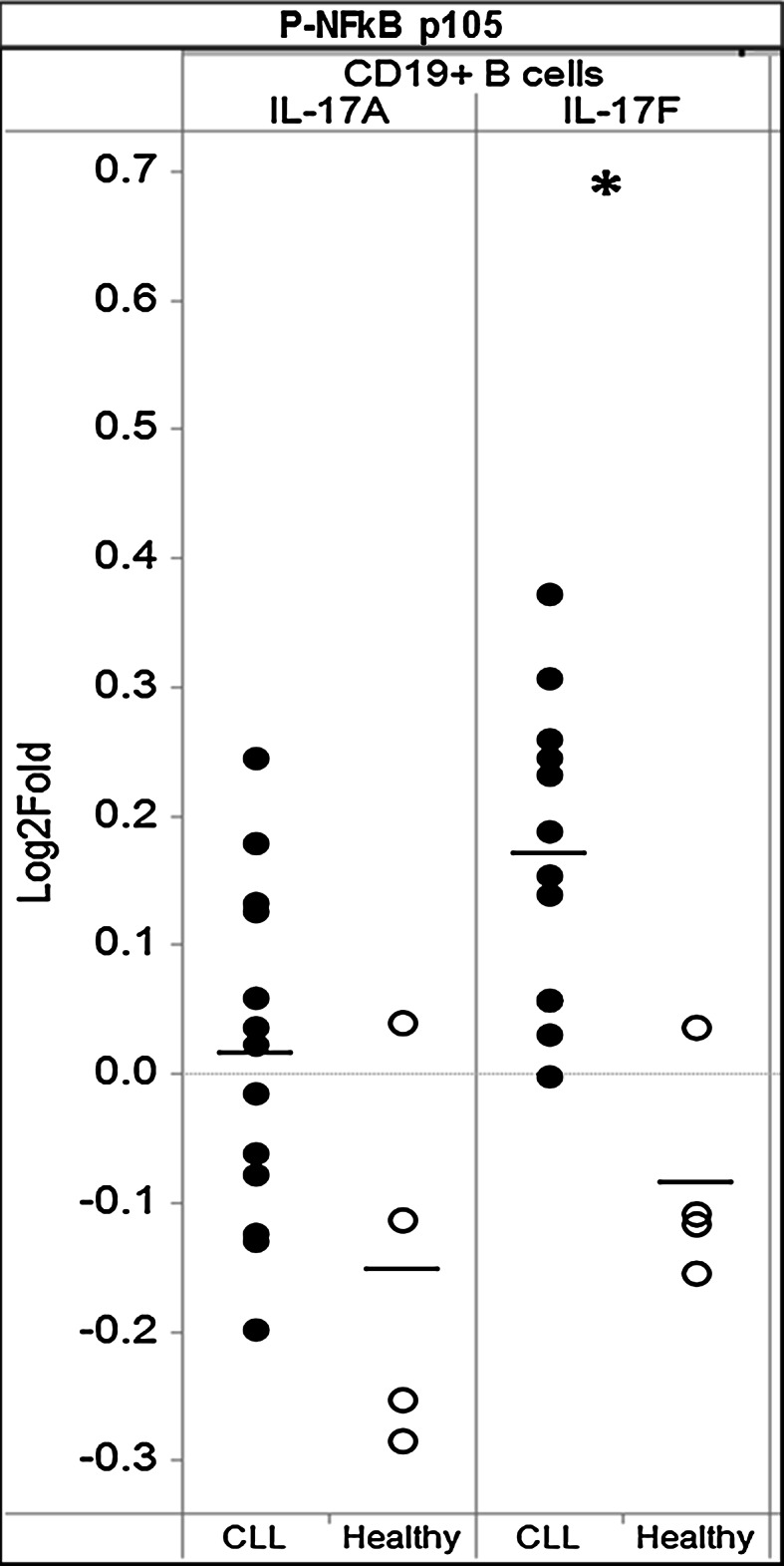
IL-17F triggers NFκB signaling in CLL, but not in healthy B cells. **a** CLL and healthy PBMCs were stimulated with IL-17F (20 ng/ml) or IL-17A (20 ng/ml) for 5 min. Phosphorylation of NFκB p105 was examined on CD19^+^ B cells by calculating the fold change in mean fluorescence intensity (MFI) for treated cells as compared to untreated cells for each donor sample. *Each circular dot* represents an individual CLL patient or healthy donor in which there were at least 100 gated CD19^+^ cells. A similar analysis was performed for ERK (T202/Y204) and Akt (S473) (data not shown). Statistical significance between CLL and healthy was examined by unpaired two-sided Student’s *t* test (*p < 0.05)

To begin to address the functional implications of the presence of elevated levels of IL-17F in CLL, we analyzed the responsiveness of CLL (*n* = 13) and age-matched healthy PBMCs (*n* = 4) to IL-17F and IL-17A using a SCNP approach. A modest increase in activation of the canonical pNFkBp105 pathway was observed in CLL B cells but not healthy donor B cells following IL-17F modulation (Fig. 5), the difference between the two donor populations being significant (*p* < 0.05) as measured by the two-sided Student’s *t* test. A weak increase in pNFkBp105 pathway signaling above baseline was observed in response to IL-17A for CLL B cells; however, the difference between CLL and healthy donor B cell signaling was not significant. These data highlight the activation of distinct IL-17 signaling pathways in CLL. IL-17-modulated signaling appeared to be specific to the NFkB pathway in this study, as there was no activation of pAkt or pERK after either IL-17F or IL-17A modulation (data not shown).

Although significant differences in IL-17F-modulated signaling between CLL and healthy B cells were identified, analysis of receptor expression levels in a subset of donors suggested that IL-17RA and IL-17RC receptor expressions were comparable between CLL and healthy B cells (data not shown). Thus, this analysis of modulated signaling indicates that the identified functional alterations were not revealed by surface phenotyping and were not due to differences in levels of expression of receptor molecules for the IL-17 ligands, but instead resulted from altered functional signaling capacity in the B-CLL cells.

### IL-17F triggers increased NFkB signaling in CLL T cells

**Fig. 6:**
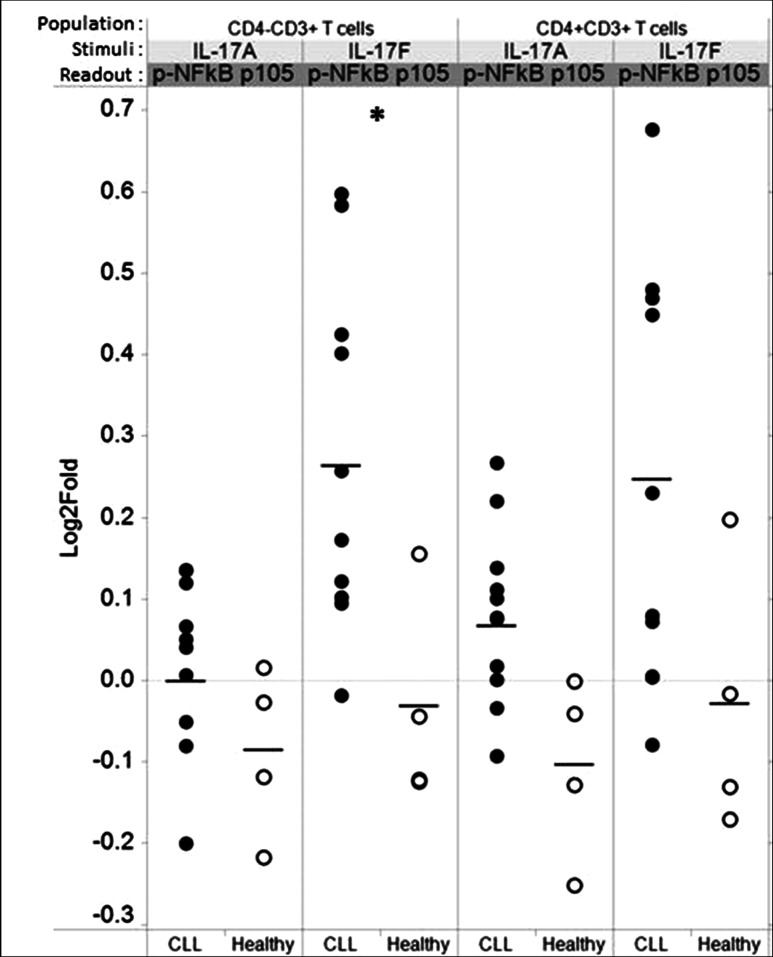
IL-17F triggers NFκB signaling in CLL, but not in healthy CD4^−^CD3^+^ (CD8^+^) cells. **a** CLL and healthy PBMCs were stimulated with IL-17F (20 ng/ml) or IL-17A (20 ng/ml) for 5 min. Phosphorylation of NFκB p105 was examined on CD4^+^CD3^+^ and CD4^−^CD3^+^ T cells by calculating the fold change in MFI for the cells in the IL-17F modulated as compared to the unmodulated (basal control) well for each donor sample. *Each circular dot* represents an individual CLL patient or healthy donor in which there were at least 100 gated cells. A similar analysis was performed for ERK (T202/Y204) and Akt (S473) (data not shown). Statistical significance between CLL and healthy was examined by unpaired two-sided Student’s *t* test (*p < 0.05)

We also examined signaling cascades triggered by IL-17F and IL-17A in T cells from the same cohort of 13 CLL patients and four healthy control subjects. IL-17F triggered a modest increase in activation of the canonical pNFkBp105 pathway in CLL donor CD4^−^CD3^+^T (CD8) cells and CD4^+^CD3^+^ T cells (Fig. 6) but no activation of signaling in healthy donor T cells. In contrast to IL-17F, IL-17A induced weak to no activation of the canonical pNFkBp105 pathway in either population of T cells (CD4^−^CD3^+^ and CD4^+^CD3^+^) in CLL and healthy donor samples. These data indicate potentially different functions for the two cytokines in CLL and a difference in IL-17F responsiveness between CLL and healthy donor T cells. As in CLL B cells, no IL-17F → pAKT or pErk signaling was observed.

### Dysregulation of signaling by Th17-inducing factors may enhance Th17F cell differentiation

**Fig. 7:**
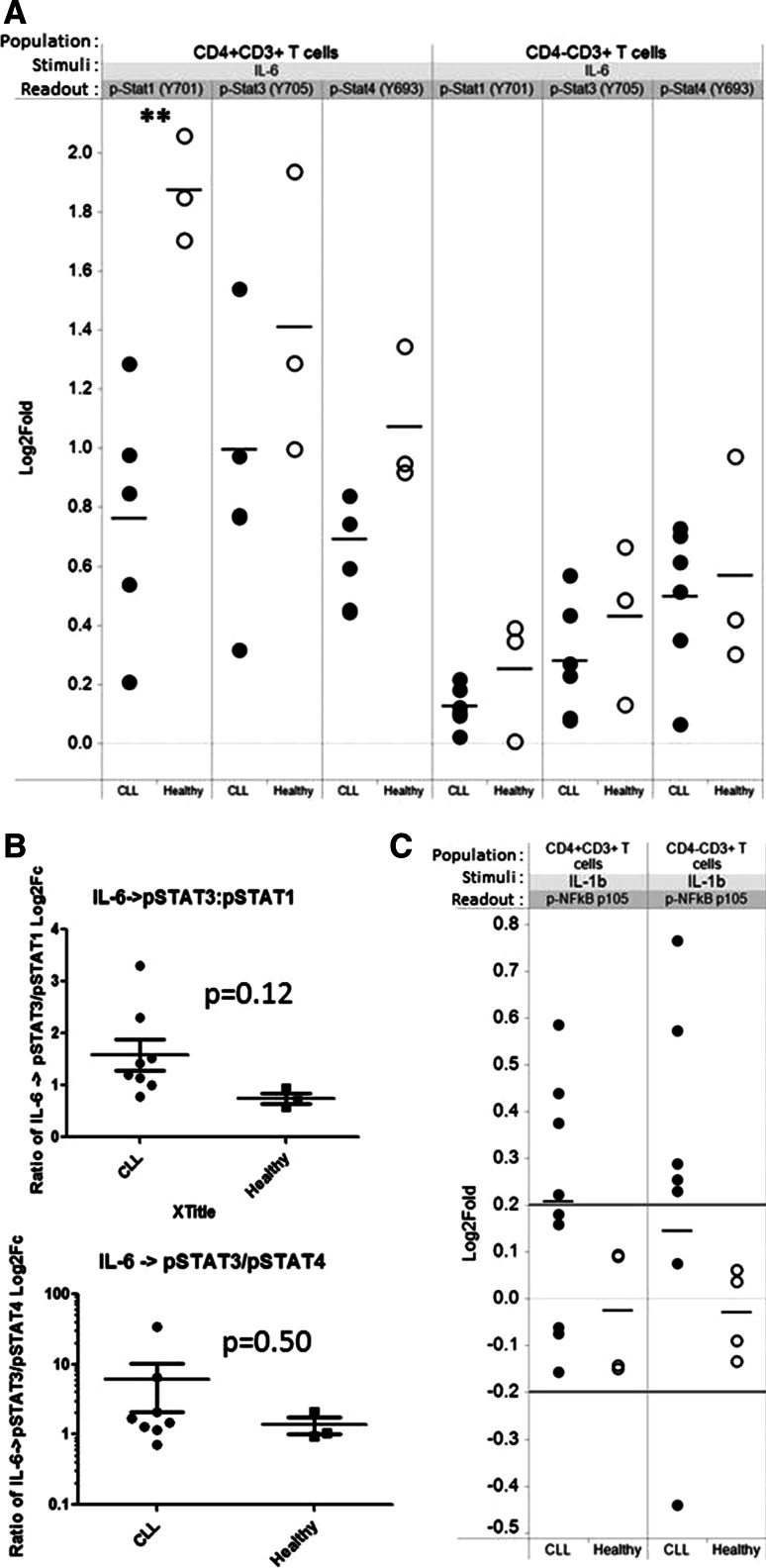
Dysregulation of signaling by Th17-inducing factors may enhance Th17F cell differentiation**. a**, **b** CLL and healthy PBMCs were stimulated with IL-6 (50 ng/ml) (**a**) or IL-1β (100 ng/ml) **(c)** for 5 min. Phosphorylation of STAT1, STAT3, STAT4, or NF-kBp105 was examined on CD4^+^CD3^+^ and CD4^−^CD3^+^ T cells, by calculating the fold change in MFI for the cells in the modulated as compared to the unmodulated (basal control) wells for each donor sample. **b** For CD4^+^CD3^+^ T cells, the ratio of IL-6-induced pSTAT3 to pSTAT1, as well as the ratio of IL-6-induced pSTAT3 to pSTAT4, was calculated for each donor sample. *Each circular dot* represents an individual CLL patient or healthy donor in which there were at least 100 gated CD4^+^CD3^+^ or CD4^−^CD3^+^ cells. Statistical significance was examined by unpaired Student’s *t* test (*p < 0.05; **p < 0.005)

Because the differentiation and expansion of Th17 cells are dependent upon signals supplied by the cytokines IL-6 and IL-1β, among others [36–38], we next analyzed signaling downstream of IL-6 (measured via p-STAT1/3/4) and IL-1β (measured via pNFkBp105) in CD4^+^CD3^+^ and CD4^−^CD3^+^ T cells from CLL patients and healthy controls. For both CLL and healthy T cells, treatment with IL-6 induced greater phosphorylation of STAT3 and STAT1 in CD4^+^CD3^+^ as compared to CD4^−^CD3^+^ (CD8) T cells (Fig. 7a). Of note, STAT1 phosphorylation was significantly higher in healthy versus CLL CD4^+^CD3^+^ T cells (*p* < 0.005), while the levels of STAT3 phosphorylation were overall higher in healthy but were not significantly different. Further, the ratios of IL-6-induced pSTAT3 to pSTAT1 in CD4^+^CD3^+^ T cells trended higher for CLL than healthy, although the differences were not statistically significant (Fig. 7b). By comparison the ratio of pSTAT3 to pSTAT4, while also higher in CLL than healthy, was less marked. Upon IL-1β modulation, both CLL CD4^+^CD3^+^ and CD4^−^CD3^+^ T cells displayed greater phosphorylation of NFkBp105 than healthy (Fig. 7c).

## Discussion

CLL is the most common adult leukemia in the USA, and despite extensive clinical and basic research, the disease remains incurable. It is clear that the cellular and cytokine microenvironment surrounding leukemic B cells greatly influences the growth and survival of the clone, providing pro- and antitumor signals [3]. T cell dysregulation is a common feature of CLL, characterized by impaired immune synapse formation and altered T-subset balance [6–8, 13]. The role of T cell subset balance in host antitumor immune responses is complex and incompletely understood, with specific subsets having variable and at times opposite effects in the setting of different tumors. We recently identified a previously unrecognized correlation between the absolute number of circulating Th17s and clinical outcome in CLL [18]. We further showed that the Th17-promoting cytokines IL-6 and IL-1 are elevated in a subset of CLL patients and are members of a cluster of cytokines whose presence correlates with longer TTFT and longer overall survival in this disease [26]. These findings suggested for the first time that Th17s and cytokines that promote the development of Th17s favor better clinical outcome.

The Th17/IL-17 axis can play pro- and antitumor roles depending upon the type of malignancy studied [21–25]. The underlying basis for this apparent discrepancy may reflect differences between the tumors themselves, but may also reflect the pleiotropic nature of Th17 actions, some of which promote (e.g., angiogenesis) and others inhibit (e.g., CTL activation) tumor growth. The wide range of Th17 actions is presumably a result of the fact that this T cell subset produces multiple cytokines in addition to IL-17A, including IL-17F, IL-22, IL-21, IL-10 IL-9, and CCL20, each of which has a unique profile of biological functions. In the current study, we analyzed the expression of IL-17F in CD4^+^ T cells from CLL patients and age-matched healthy donors. We report that both circulating (Fig. 1b) and in vitro differentiated (Fig. 3a, b) Th17 cells express higher levels of IL-17F than similarly isolated or in vitro differentiated Th17 cells from healthy age-matched individuals, although the difference was statistically significant only in the case of Th17 cells activated for 7 days in vitro in the presence of Th17-promoting cytokines. It is of interest to note that circulating levels of IL-17F-expressing CD4^+^ T cells appear to fall into two discrete clusters, one displaying a relatively high percentage of IL-17F-expressing CD4^+^ T cells and the other displaying levels comparable to, or even lower than, those observed for age-matched healthy donors (Fig. 1b). Studies are currently underway to determine whether these clusters correlate with CLL prognostic markers (e.g., mutation status, CD38 expression) or clinical status (overall survival, TTFT).

IL-17F is highly homologous to IL-17A both structurally and functionally, and the two cytokines bind to the same receptor, albeit with different affinities [27], but there is increasing evidence that the two have discrete functions as well [27–30]. Therefore, it is of considerable interest that although the levels of IL-17F-expressing CD4^+^ T cells are higher in CLL versus healthy PBMCs following in vitro stimulation for 7 days in the presence of Th17-promoting cytokines, the percent of IL-17A-expressing CD4^+^ T cells are not (data not shown). This suggests that the balance of cytokines secreted by CLL Th17 cells differentiated within the CLL cellular microenvironment (mimicked in our PBMC cultures) may differ from that observed in Th17 cells differentiated within a “non-CLL” cellular microenvironment (IL-17F higher relative to IL-17A in Th17 cells differentiated within CLL milieu). Since there are differences in receptor binding affinity, signaling, and bioactivity profiles between IL-17F and IL-17A, the functional program of Th17s in CLL tissue microenvironments might be distinct from that seen in healthy subjects.

Th17 cells are generated from naïve T cells when they are activated in the presence of a select set of cytokines that include IL-1β, IL-6, IL-21, IL-23, and TGFβ. The mechanisms that regulate Th17 differentiation in the context of CLL are unknown, although the Th17-inducing cytokines IL-1β and IL-6 are elevated in a subset of CLL patients with good prognosis [26] and presumably drive Th17 differentiation in that population. Herein, we report that IL-23, a cytokine critical for Th17 cell maintenance and expansion, is also significantly elevated in serum from CLL patients as a whole compared to age-matched healthy controls (Fig. 2). This finding suggests a potential mechanism that may contribute to the higher circulating levels of CD4^+^ T cells expressing IL-17F (this study) and IL-17A [18] observed in CLL patients. It is interesting to note that we observed considerable heterogeneity in serum IL-23 levels among CLL patients. It remains to be determined whether serum IL-23 levels correlate with specific prognostic markers (e.g., mutation status, CD38 expression) or clinical status (overall survival, TTFT). Under normal conditions, the relative levels of p-STAT proteins within lymphocytes are believed to control T cell differentiation, with the ratio of p-STAT3:p-STAT1 being a critical thermostat in the Th17 differentiation axis, a higher p-STAT3:p-STAT1 favoring Th17 generation [39]. Therefore, our finding that IL-6-stimulated CD4^+^ T cells from CLL patients displayed a higher ratio of p-STAT3, a pro-Th17 factor, relative to p-STAT1, an inhibitory Th17 factor, than CD4 + T cells from healthy subjects (Fig. 7), raises the possibility that CD4^+^ T cells from CLL patients, or a subset of CLL patients, are “primed” to differentiate along the Th17 lineage in response to activation signals.

A growing body of literature supports the existence of multiple forms of Th17 cells expressing different profiles of cytokines and mediating different biological functions. The CLL-specific microenvironmental triggers that induce the generation of a population of Th17 cells with unique secretory properties (higher levels of IL-17F relative to IL17A) are as yet unknown. Our finding that the coculture of CLL B cells with autologous CD4^+^ T cells leads to higher percentages of IL-17F-expressing Th17 cells (Fig. 4) suggests that the CLL B cell itself, in certain contexts, has the capacity to trigger a signaling pathway(s) in the T cell that drives Th17 differentiation or expansion. Of potential relevance is a recent report demonstrating that CD5 co-stimulation induces stable Th17 development in vitro by promoting IL-23R expression and sustained STAT3 activation [40]. In humans, there is no confirmed ligand for CD5, but there is evidence that CD72, a C-type lectin, may interact with CD5 or that CD5 may be homophilic, permitting binding of CD5 on the surface of adjacent cells [41]. Moreover, there is another, potentially complementary role that CLL B cells might be playing. Naïve T cells do not express the IL-23R, which would make them unresponsive to this critical Th17-inducing cytokine signal [42]. CD5, which is highly expressed on CLL, but not healthy B cells, has been reported to induce IL-23R in naïve cells. This suggests a second mechanism, whereby the CLL B cell could enhance IL-17-expressing Th17 generation in our coculture system—upregulation of IL-23R on naïve CD4^+^ T cells via homotypic interactions through CD5, and then supplying the IL-23. At this point, the increased levels of IL-23 in the serum of CLL patients (Fig. 2) have not been shown to derive directly from the CLL B cell, although results from our coculture studies could be interpreted as CLL B cells partially supplementing the requirement for IL-23 in the Th17 cocktail (Fig. 4). The extent that CLL B cells or another cell in the CLL microenvironment selectively promotes IL-17F (or IL-17A) production remains to be explored.

To begin to interrogate the signaling interplay of high IL-17F-expressing Th17 cells with CLL cells and the tumor microenvironment, we performed SCNP analyses in which PBMC samples from CLL patients and healthy donors were incubated in vitro with IL-17F and the evoked signaling in pathways known to be triggered by IL-17F, including NFκB (p105NFkB), PI3K (p-Akt), and MAPK (p-ERK), was quantified. Surprisingly, we found that IL-17F (but not IL-17A) stimulated NFκB activation in CLL B cells but not B cells from healthy subjects (Fig. 5). Leukemic B cells from CLL patients studied immediately ex vivo often exhibit high levels of activated NFκB [43], although this can differ among individual subjects [44]. For those with elevated active NFκB, this finding suggests either constitutive activation or ongoing stimulation of the pathway in vivo. Multiple mechanisms, including BCR engagement, have been reported to activate NFκB in the context of CLL [45]. Our finding that IL-17F triggers NFκB signaling in CLL B cells identifies another potential mechanism within the CLL microenvironment that may mediate NFκB activation. Furthermore, the fact that stimulation of NFκB signaling was accomplished only by IL-17F, not IL-17A and occurred only in CLL B cells and not those from healthy donors is provocative, and the clinical association of this interaction is under study. Finally, the inability to detect a robust signaling event after IL-17A exposure, as measured by signaling through NFκB, Akt, or Erk activation, highlights the unique actions of IL-17F in the disease.

In addition, our finding that IL-17F also triggers NFkB activation in CD4^−^CD3^+^ (CD8) cells, but not CD4^+^CD3^+^ T cells (Fig. 6), is particularly intriguing. The role of Th17s in antitumor immunity remains controversial, with some studies demonstrating pro-tumor and other studies antitumor effects. This has been presumed to reflect the at times opposing functions of the cytokines produced by Th17 cells. IL-17A produced by Th17 cells promotes tumor growth by inducing angiogenesis, while IL-21 produced by Th17 cells mediates antitumor effects including induction of apoptosis. Our finding that IL-17F, another cytokine variably produced by Th17 cells, activates the NFkB signaling cascade in CD8 T cells, which are known to play an important antitumor role, suggests an additional, antitumor mechanism—the recruitment and/or activation of antitumor immune cells. In order to be able to evaluate the clinical influence of Th17 cells in a particular clinical situation, it is necessary to evaluate the profile of cytokines released by endogenous Th17 cells. One could envision that in patients with the same disease, differences within the microenvironment could modulate the types of cytokines secreted by Th17 cells (and other T cell subsets), thereby differentially influencing outcome.

In summary, our studies have demonstrated that the levels of IL-17F-expressing CD4^+^ T cells are significantly higher in CLL versus healthy PBMCs following in vitro stimulation in the presence of Th17-promoting cytokines and that both leukemic B cells and CLL T cells are responsive to IL-17F-mediated signaling, specifically activating NFkB. Taken together, our data suggest that in tissues where most activation and division occur, the functional program of Th17s in CLL might be distinct from that seen in healthy subjects, favoring IL-17F production and responsiveness. It remains unclear at this point whether the effects of IL-17F on cells in the leukemic microenvironment are capable of mediating tumor-promoting or tumor-suppressing actions.

## Electronic supplementary material

Supplementary material 1 (DOCX 25 kb)

## Abbreviations

BCR: B cell antigen receptor
CLL: Chronic Lymphocytic Leukemia
DMSO: Dimethylsulfoxide
IL-17: Interleukin-17
M-CLL: *IGHV*-mutated CLL
U-CLL: *IGHV*-unmutated CLL
MFI: Mean fluorescence intensity
mAb: Monoclonal antibody
NFkB: Nuclear factor kappa-light-chain-enhancer of activated B cells
NHS: Normal human serum
PBMCs: Peripheral blood mononuclear cells
SCNP: Single-cell network profiling
TTFT: Time-to-first treatment
TGF-β1: Transforming growth factor β
