# Supplementary material for: Identification and characterization of distinct IL-17F expression patterns and signaling pathways in chronic lymphocytic leukemia and normal B lymphocytes
Source: Immunol Res. 2015 Oct 19;63(1-3):216–27. doi: 10.1007/s12026-015-8722-5 (PMC4648985; doi:10.1007/s12026-015-8722-5)
Supplement: Supplementary file 1 — Supplementary material 1 (DOCX 25 kb) [file 12026_2015_8722_MOESM1_ESM.docx]

**Suppl. Table 1. Clinical & laboratory characteristics of CLL patients and healthy subjects.**

| **# Code** | **CLL vs Healthy** | **Gender** | **IGHV**  **Mutation Status** | **CD38 Level** | **Fluorescence in situ hybridization (FISH)** | **Cells used for: (IC, IL-23 ELISA, T±B co-culture, SCNP/signaling)** |
| --- | --- | --- | --- | --- | --- | --- |
| **0692** | CLL | Female | M | High | Trisomy 12 | IC / baseline & 7d, IL‑23 ELISA, SCNP |
| **1086** | CLL | Male | U | Low | del13q | IC / baseline & 7d |
| **1734** | CLL | Female | M | NA | del13q | IC / baseline & 7d |
| **1851** | CLL | Male | M | Low | Trisomy 12 | IC / baseline & 7d |
| **2049** | CLL | Female | NA | NA | NA | IC / baseline & 7d |
| **0514** | CLL | Male | NA | Low | NA | IC / baseline & 7d |
| **1090** | CLL | Male | M | Low | Normal | IC / baseline & 7d, IL‑23 ELISA |
| **0710** | CLL | Male | M | High | Normal | IC / baseline & 7d, IL‑23 ELISA |
| **0321** | CLL | Male | U | Low | Trisomy 12 | IC / baseline & 7d |
| **1381** | CLL | Female | M | Low | NA | IC / baseline & 7d |
| **0700** | CLL | Male | M | Low | del 13q | IC / baseline & 7d, IL‑23 ELISA, SCNP |
| **1380** | CLL | Male | U | Low | del13q | IC / baseline & 7d |
| **0712** | CLL | Male | M | Low | NA | IC / baseline & 7d, IL‑23 ELISA |
| **0699** | CLL | Male | M | Low | del13q | IC / baseline & 7d, IL‑23 ELISA |
| **0394** | CLL | Male | U | High | normal | IC / baseline & 7d |
| **0230** | CLL | Female | U | Low | NA | IC / baseline & 7d |
| **0569** | CLL | Male | U | High | Normal | IL-23 ELISA |
| **1278** | CLL | Male | U | High | Trisomy 12 | IL-23 ELISA |
| **1013** | CLL | Male | U | High | Trisomy 12 | IL-23 ELISA |
| **1156** | CLL | Male | U | High | del13q,del17p | IL-23 ELISA |
| **1167** | CLL | Male | M | Low | del13q | IL-23 ELISA |
| **1153** | CLL | Male | U | High | Normal | IL-23 ELISA |
| **1016** | CLL | Male | U | High | NA | IL-23 ELISA |
| **0705** | CLL | Female | U | High | del17p,del13q | IC / baseline, IL-23 ELISA |
| **1028** | CLL | Male | M | Low | NA | IL-23 ELISA |
| **0721** | CLL | Male | U | Low | Trisomy 12 | IC / baseline, IL-23 ELISA |
| **0678** | CLL | Female | M | Low | NA | IL-23 ELISA |
| **0896** | CLL | Female | U | High | NA | IL-23 ELISA |
| **1168** | CLL | Male | M | Low | del13q | IL-23 ELISA |
| **0726** | CLL | Male | M | High | del13 q | IL-23 ELISA |
| **0724** | CLL | Female | M | Low | del13 q | IL-23 ELISA |
| **0276** | CLL | Male | M | Low | del13q | IL-23 ELISA |
| **0439** | CLL | Male | U | Low | del6q,Trisomy 12 | IL-23 ELISA, SCNP |
| **0701** | CLL | Male | U | High | Normal | IL-23 ELISA |
| **0196** | CLL | Male | M | NA | Trisomy 12 | IL-23 ELISA |
| **1459** | CLL | Female | NA | NA | del13q | IL-23 ELISA |
| **1417** | CLL | Female | M | NA | Normal | IL-23 ELISA |
| **1279** | CLL | Male | U | Low | del 13q | IL-23 ELISA |
| **1280** | CLL | Male | U | High | Normal | IL-23 ELISA |
| **1440** | CLL | Male | U | Low | Normal | IL-23 ELISA |
| **0493** | CLL | Male | U | Low | del13q | IL-23 ELISA |
| **1453** | CLL | Male | M | NA | NA | IL-23 ELISA |
| **0675** | CLL | Female | U | High | del11q, del13q | IL-23 ELISA |
| **0989** | CLL | Male | M | NA | Normal | IL-23 ELISA |
| **0950** | CLL | Male | U | Low | del13q | IL-23 ELISA |
| **1460** | CLL | Female | M | Low | Normal | IL-23 ELISA |
| **0275** | CLL | Female | M | NA | del13q | IL-23 ELISA |
| **1454** | CLL | Female | U | NA | Normal | IL-23 ELISA |
| **1288** | CLL | Female | U | NA | NA | IL-23 ELISA |
| **1300** | CLL | Male | M | High | Normal | IL-23 ELISA |
| **1277** | CLL | Male | U | Low | Normal | IL-23 ELISA |
| **0991** | CLL | Male | U | High | del11q, del13q | IL-23 ELISA |
| **0616** | CLL | Male | M | Low | del13q | IL-23 ELISA |
| **1291** | CLL | Male | M | Low | Normal | IL-23 ELISA |
| **0609** | CLL | Male | U | Low | Normal | IL-23 ELISA |
| **1445** | CLL | Male | U | High | NA | IL-23 ELISA |
| **0900** | CLL | Male | U | High | Normal | IL-23 ELISA |
| **0280** | CLL | Male | M | Low | del13q | IL-23 ELISA |
| **0444** | CLL | Male | M | High | Normal | IL-23 ELISA |
| **0618** | CLL | Female | M | NA | Del13q,Trisomy12 | IL-23 ELISA |
| **0314** | CLL | Male | NA | NA | NA | IL-23 ELISA |
| **0711** | CLL | Male | M | Low | Normal | IL-23 ELISA |
| **0748** | CLL | Male | M | Low | del13q | IL-23 ELISA |
| **0949** | CLL | Male | M | Low | del11q | IL-23 ELISA |
| **0803** | CLL | Female | NA | NA | Normal | IL-23 ELISA |
| **0409** | CLL | Male | M | NA | Del13q | IL-23 ELISA |
| **0163** | CLL | Male | M | High | del6q, del11q, del13q | IL-23 ELISA |
| **1081** | CLL | Female | U | High | del11q, del13q | T±B co-culture |
| **1388** | CLL | Male | M | Low | NA | T±B co-culture |
| **1124** | CLL | Female | M | High | Del13q | T±B co-culture |
| **0485** | CLL | Female | M | High | Normal | SCNP |
| **0854** | CLL | Male | M | Low | del13q | SCNP |
| **1643** | CLL | Female | U | Low | del13q | SCNP |
| **0910** | CLL | Male | M | Low | del13q | SCNP |
| **0921** | CLL | Male | U | Low | NA | SCNP |
| **0934** | CLL | Female | U | Low | Trisomy 12 | SCNP |
| **1180** | CLL | Female | M | Low | Normal | SCNP |
| **0834** | CLL | Female | U | Low | del13q | SCNP |
| **0879** | CLL | Male | M | Low | Normal | SCNP |
| **0781** | CLL | Male | M | High | NA | SCNP |
| **17H** | Healthy | NA |  |  |  | IC / baseline & 7d |
| **18H** | Healthy | NA |  |  |  | IC / baseline & 7d |
| **18H** | Healthy | NA |  |  |  | IC / baseline & 7d |
| **22H** | Healthy | NA |  |  |  | IC / baseline & 7d |
| **22H** | Healthy | NA |  |  |  | IC / baseline & 7d |
| **D850** | Healthy | NA |  |  |  | IC / baseline & 7d |
| **D852** | Healthy | NA |  |  |  | IC / baseline & 7d |
| **D846** | Healthy | NA |  |  |  | IC / baseline & 7d |
| **D847** | Healthy | NA |  |  |  | IC / baseline & 7d |
| **AG434b** | Healthy | NA |  |  |  | IL-23 ELISA |
| **AG430** | Healthy | NA |  |  |  | IL-23 ELISA |
| **AG429a** | Healthy | NA |  |  |  | IL-23 ELISA |
| **AG428** | Healthy | NA |  |  |  | IL-23 ELISA |
| **AG427** | Healthy | NA |  |  |  | IL-23 ELISA |
| **AG426** | Healthy | NA |  |  |  | IL-23 ELISA |
| **AG418** | Healthy | NA |  |  |  | IL-23 ELISA |
| **AG416** | Healthy | NA |  |  |  | IL-23 ELISA |
| **AG415** | Healthy | NA |  |  |  | IL-23 ELISA |
| **AG380** | Healthy | NA |  |  |  | IL-23 ELISA |
| **AG327** | Healthy | NA |  |  |  | IL-23 ELISA |
| **AG325** | Healthy | NA |  |  |  | IL-23 ELISA |
| **AG235** | Healthy | NA |  |  |  | IL-23 ELISA |
| **AG323** | Healthy | NA |  |  |  | IL-23 ELISA |
| **AG500** | Healthy | NA |  |  |  | IL-23 ELISA |
| **H.01** | Healthy | Male |  |  |  | SCNP |
| **H.02** | Healthy | Male |  |  |  | SCNP |
| **H.03** | Healthy | Female |  |  |  | SCNP |
| **H.04** | Healthy | Female |  |  |  | SCNP |
